# Ceramide as a Target of Marine Triterpene Glycosides for Treatment of Human Myeloid Leukemia

**DOI:** 10.3390/md14110205

**Published:** 2016-11-03

**Authors:** Seong-Hoon Yun, Sung-Won Shin, Valentin A. Stonik, Joo-In Park

**Affiliations:** 1 Department of Biochemistry, Dong-A University College of Medicine, 32 Daesingongwon-ro, Seo-Gu, Busan 49201, Korea; tpohot10@nate.com (S.-H.Y.); lunaticblue@lycos.co.kr (S.-W.S.); 2 G.B. Elyakov Pacific Institute of Bioorganic Chemistry, Far-Eastern Branch of the Russian Academy of Science, Vladivostok 690022, Russia; stonik@piboc.dvo.ru; 3 Department of Bioorganic Chemistry and Biotechnology, School of Natural Sciences, Far East Federal University, Vladivostok 690091, Russia

**Keywords:** stichoposides, anti-leukemic activity, ceramide, sphingomyelinase, ceramide synthase

## Abstract

Acute myeloid leukemia (AML) is a heterogeneous myeloid clonal disorder exhibiting the accumulation of immature myeloid progenitors in the bone marrow and peripheral blood. Standard AML therapy requires intensive combination chemotherapy, which leads to significant treatment-related toxicity. The search for new, low toxic marine agents, inducing the generation of ceramide in leukemic cells is a new approach to improve the therapy of leukemia. This review focuses on the metabolism of sphingolipids, the role of ceramide in treating leukemia, and the antitumor activity, related to ceramide metabolism, of some marine metabolites, particularly stichoposides, triterpene glycosides extracted from sea cucumbers of the family Stichopodiidae.

## 1. Introduction

Sphingolipids have been recognized as bioactive lipids involved in the regulation of various cell functions, including cell death, proliferation/survival, autophagy, migration, secretion and immunity [1,2,3]. About two decades ago, it was first reported that ceramide induced cell differentiation and death in human leukemia HL-60 cells [4,5]. The subcellular compartmentalization of active ceramide and the putative function among ceramide molecular species have been investigated in several kinds of cancers [6,7].

Several marine metabolites are known to induce the generation of ceramide in tumor cells, including triterpene glycosides, which are widely distributed in plants and also found in marine invertebrates [8]. Many marine triterpene glycosides are good sources for developing anticancer agents because of low toxicity suitable for administration, promising activities and wide diversity in their mechanisms of action. Stichoposide C (STC) and stichoposide D (STD) are marine triterpene glycosides isolated from sea cucumbers *Stichopus chloronotus* [9,10], *Thelenota ananas* [11], and *Thelenota anax* [12]. STC and STD have the same aglycone but different sugar compositions; STC contains a quinovose, while STD contains a glucose as the second monosaccharide unit.

This review highlights our current understanding of the metabolism of sphingolipids, the tumor suppressive functions of ceramide, and the action mechanisms of stichoposides related to ceramide metabolism in treating leukemia. Some data on other marine inducers of ceramide accumulation in tumor cells are also given.

## 2. Metabolism of Sphingolipids

Sphingolipids are structural components of membrane lipids and also involved in mediating a variety of intracellular functions [1,2,3]. Synthesis and degradation of sphingolipids are important for cellular homeostasis and various enzymes are included in their metabolism. We describe these enzymes and their reactions. Their responsible genes, biochemical characteristics, subcellular localization and regulation were summarized by Kitatani et al. [13].

### 2.1. De Novo Synthesis of Sphingolipids

**Figure 1:**
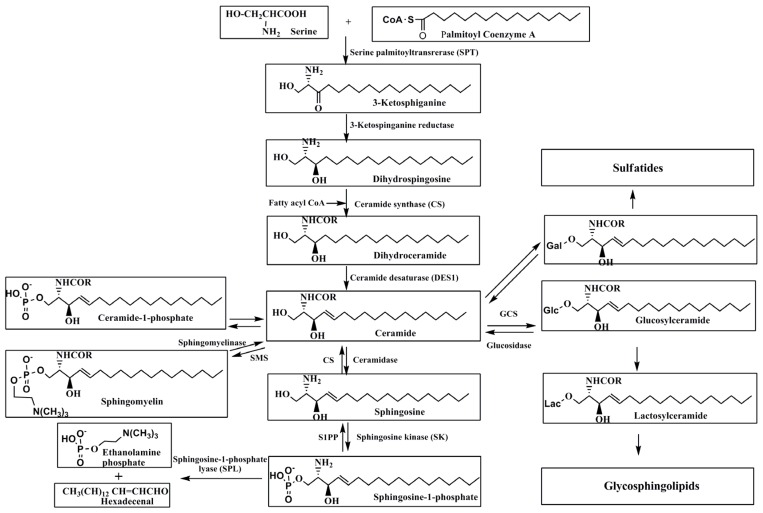
Synthesis and degradation of sphingolipids. GCS; glucosylceramide synthase, SMS; sphingomyelinsynthase, S1PP; sphingosine-1-phosphate phosphatase, Gal; galactose, Glc; glucose, Lac; lactose.

The metabolic pathways of de novo synthesis of sphingolipids are shown in Figure 1. De novo synthesis of sphingolipids begins at the endoplasmic reticulum with the condensation of palmitoyl-CoA and serine by serine palmitoyl transferase (SPT) [14,15], generating 3-ketosphinganine. This is converted to dihydrosphingosine by a 3-ketosphinganine reductase. Dihydrosphingosine can be acylated by a family of ceramide synthases (CerS), thereby giving rise to the formation of various dihydroceramides. At present, six different CerS isoforms have been identified [16]. Different kinds of CerS produce various ceramide species with distinct chain lengths of fatty acids [17]. CerS1 primarily generates C18-ceramide. CerS2 synthesizes ceramide containing C20–C26 fatty acids, with little or no synthesis of C16-ceramide or C18-ceramide [18]. CerS3 synthesizes C24-ceramide and ceramides with longer acyl chains. CerS4 synthesizes ceramides containing C18-22 fatty acids. CerS5 and CerS6 synthesize C14- and C16-ceramide [19]. Ceramide desaturase (DES1) [20] catalyzes the synthesis of ceramide from dihydroceramide, which is the last step for the de novo synthesis of ceramide. These reactions occur in endoplasmic reticulum, and ceramide acts as a building block for most of sphingolipid species. Transport of ceramide by ceramide transfer protein (CERT) [14,21] and/or other transporting protein (s) to the Golgi is required for the synthesis of ceramide-1-phosphate, sphingomyelin (SM), galactosylceramide, and glucosylceramide. The glycolipids are further metabolized to complex sphingolipids. Ceramide transported to Golgi by CERT is converted to SM by SM synthase (SMS), and then SM is distributed to plasma membranes and functions as a component of lipid microdomains.

### 2.2. Degradation of Sphingolipids

The degradation pathways of sphingolipids are also summarized in Figure 1. Sphingolipid catabolizing enzymes are largely localized in endolysosomes, resulting in the formation of lysosomal ceramide [22,23]. Lysosomal ceramide is formed from glucosylceramide by lysosomal acid-β-glucosidase [24,25]. It is further catabolized to sphingosine by a family of pH-dependent ceramidases [26]. This sphingolipid backbone sphingosine is used to generate ceramide through the ceramide synthase at endoplasmic reticulum. This is called the “salvage pathway” of sphingolipid synthesis [27,28]. Alternatively, sphingosine is phosphorylated by sphingosine kinase (SK) [29], forming sphingosine 1 phosphate (S1P) that is also degraded or dephosphorylated by sphingosine 1 phosphate lyase (SPL) [30] or S1P phosphatase [31], respectively. Ceramide is formed from SM by SMases such as acid, neutral, or alkaline SMase in various subcellular organelles.

## 3. Role of Ceramide in Leukemia

### 3.1. Induction of Cell Differentiation

The induction of cell differentiation is a useful strategy for overcoming leukemia. Thus, many investigators have focused on developing anticancer drugs targeted toward differentiation. Until now, retinoic acid and 1,25-(OH)_2_D_3_ are known as differentiation-inducing agents for acute promyelocytic leukemia [32]. Okazaki et al. first showed that ceramide generation by 1,25-(OH)_2_D_3_ may contribute to the induction of cell differentiation of HL-60 cells [5]. In addition, ceramide formed through the activation of cytosolic magnesium-independent neutral SMase by 1,25-(OH)_2_D_3_ functions as a second messenger in HL-60 differentiation [33,34]. Langmann et al. showed that lysosomal acid SMase activity was induced during monocytic differentiation of the monocytic leukemia cell line, THP-1, by 12-*O*-tetradecanoylphorbol-13-acetate (TPA) or 1,25-(OH)_2_D_3_, and that an increased expression of the acid SMase gene was mediated through SP1 and AP2 sites on the 5′-promoter region [35]. In addition, Kim et al. demonstrated that ceramide derivatives potentiated cell differentiation of HL-60 cells through phosphatidylinositol-3-kinase (PI3K), protein kinase (PKC), c-Jun N-terminal kinase (JNK), and extracellular regulated kinase (ERK) when combined with 1,25-(OH)_2_D_3_ [36]. Taken together, these suggest that ceramide may contribute to cell differentiation of leukemia cells.

### 3.2. Induction of Apoptosis

Short-chain ceramides have been known to induce apoptosis [4]. Several stress agents, including cytokines, chemotherapeutic drugs, ionizing radiation and photodynamic therapy, promote the generation of ceramide before the onset of apoptosis [37]. Several ceramide analogues have been synthesized and been shown to induce apoptosis in leukemia cells [38]. For example, thiouracil-ceramide induces apoptosis of human CEM leukemia cells through the caspase-independent pathway [39]. AD2646 and AD2687, synthetic ceramide analogs, had been shown to induce apoptosis through the caspase-dependent and -independent pathways in human Jurkat and HL-60 leukemia cells [40,41]. The molecular action mechanisms of ceramide mimics still remain poorly defined. Several potential targets have been proposed, including activation of serine/threonine kinase (e.g., PKCζ) [42], disruption of mitochondrial membrane potential, production of reactive oxygen species, and cytochrome c release [43], or a decreased level of glutathione [44]. In addition, positively charged ceramides seem to trigger mitochondrial permeabilization [45].

Ceramide appears to regulate diverse signaling pathways such as PKC, AKT, and phospholipase D [46,47,48]. It also activates specific serine/threonine protein phosphatases (ceramide-activated protein phosphatases), protein kinases (c-RAF, PKCζ, and kinase suppressor of RAS) and cathepsin D, a protease [49,50]. Several studies suggest that stress-activated protein kinases such as JNK, ERK or p38 kinase play important roles in inducing apoptosis in response to ceramide [51]. Kim et al. demonstrated that ceramide induced apoptosis through caspase activation, cytochrome c release, and Bax translocation through the activation of p38 kinase and the inhibition of Akt in HL-60 cells [52,53]. Liu et al. showed that nanoliposomal C6 ceramide induces apoptosis through the down-regulation of survivin, through the inhibition of ERK, in natural killer-large granular lymphocytic leukemic cells [54]. Nica et al. reported that C6 ceramide promoted apoptosis through caspase-8 activation and JNK activation resulting in inactivation of Mcl-1 in K562 cells [52]. Iwai et al. demonstrated that C2-ceramide induces apoptosis through the inhibition of catalase by caspase-dependent proteolysis [55]. Herr et al. showed that ceramide induces apoptosis through the up-regulation of CD95-L expression [56]. Taken together, the molecular mechanisms of ceramide-induced apoptosis are complex and dependent on the nature of ceramide and the cell type.

### 3.3. Induction of Autophagy

Ceramide was shown to promote autophagy by interfering with the class I PI3K pathway and increasing expression of an autophagy gene, beclin 1 [57] and to play a role in regulating autophagy and its associated cell death [58]. Even though the mechanism by which ceramide stimulates autophagy are not well defined, Pattingreet al. suggested that ceramide induces autophagy through dissociation of Beclin 1-Bcl-2 complex by the JNK1-dependent phosphorylation of Bcl-2 [58].

## 4. Action Mechanisms of Stichoposides Related to Ceramide Generation. Some Other Marine Natural Products with Similar Action Mechanisms

### 4.1. STC

**Figure 2:**
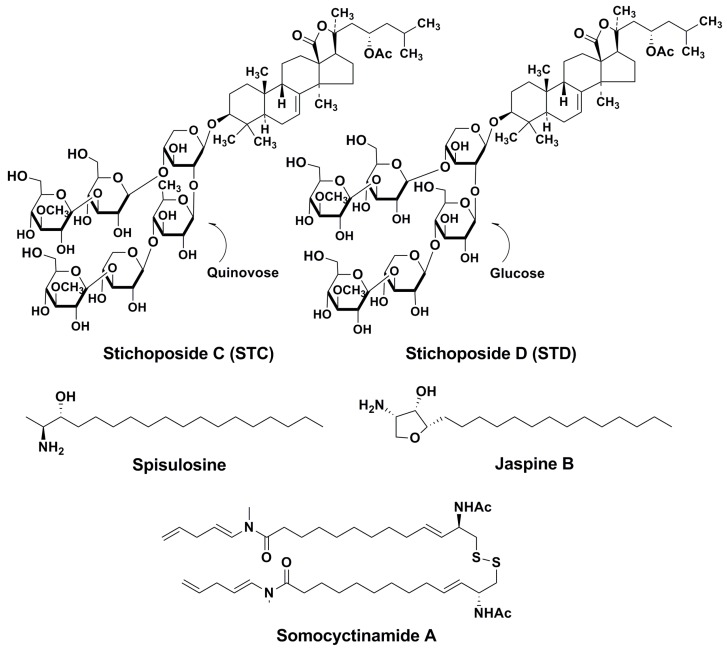
Structures of Stichoposides C and D and some other marine inducers of ceramide accumulation in tumor cells.

STC is one of main glycosides in sea cucumbers belonging to the Stichopodiidae family. It contains a quinovose as the second monosaccharide unit in the carbohydrate chain (Figure 2). A previous study suggested that the antitumor effect of STC seems to be related to its membranotropic effects [8]. However, it has been unclear about the molecular mechanisms underlying antitumor activity of STC. Our group first demonstrated that STC from *Thelenota anax* induced apoptosis of human leukemia and colorectal cancer cells through the activation of both mitochondrial and death receptor pathways [12]. Even though STC induced apoptosis of both human leukemia and colorectal cancer cells, the IC_50_ of STC in human leukemia cells (0.3–0.5 μM) was lower than that in colorectal cancer cells (2.5 μM). These results indicate that STC is an effective anticancer agent candidate in treating leukemia, even though the reasons for the differential chemosensitivity of STC in leukemia and colorectal cancer cells are still unknown. Many chemotherapeutic agents were shown to increase levels of the pro-apoptotic sphingolipid ceramide in all types of cancer cells [59].

As described in the previous sections, ceramide is generated either by de novo synthesis or by sphingomyelin hydrolysis. Ceramide is also formed by the salvage pathway [60,61,62]. Both acid and neutral SMase are involved in ceramide generation in response to apoptotic stimuli [63,64,65]. We demonstrated that STC induced apoptosis through the generation of ceramide by the activation of acid SMase following caspase-8 activation and neutral SMases following ROS generation and glutathione depletion using siRNA knockdown experiments and chemical inhibitors [12].

**Figure 3:**
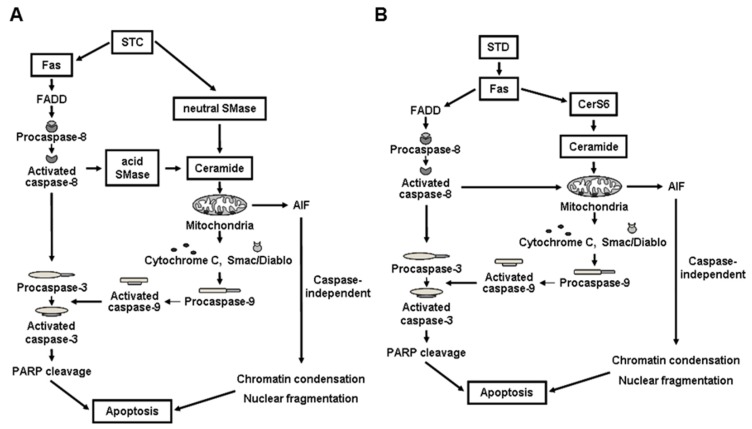
Potential molecular mechanisms of STC-induced (**A**) and STD-induced apoptosis (**B**). CerS6: ceramide synthase 6, SMase; sphingomyelinase.

The potential molecular mechanisms for STC-induced apoptosis based on our observations are shown in Figure 3A. Therefore, the target of STC appears to be acid and neutral SMase leading to increases in ceramide and apoptosis.

### 4.2. STD

STD is a related glycoside that contains glucose as the second monosaccharide unit (Figure 2). STD has shown to induce apoptosis of human leukemia cells through the activation of the death receptor pathway and mitochondrial pathway [66]. We previously observed that STC was more potent than STD in inducing cell death [66]. These results are consistent with the relative membranotropic activities of STC and STD, suggesting that their anticancer activities may be related to their membranotropic activities. More importantly, STC and STD did not have any toxicity in normal hematopoietic progenitor cells or in a mouse xenograft tumor model [12,67].

Yun et al. firstly demonstrated that STD can induce apoptosis of leukemia cells through the activation of CerS6 leading to increased ceramide levels [67]. The activation of CerS6 appears to be subsequent to the activation of the death receptor Fas (CD95) in lipid rafts by STD [67]. Furthermore, the functional importance of CerS6 in antitumor activity of STD was confirmed by CerS shRNA-knockdown stable cell xenograft models [67]. The potential molecular mechanisms for STD-induced apoptosis based on our observations are shown in Figure 3B. These results suggest that the difference in only one sugar between STC and STD may influence both the potency and the molecular mechanisms for their actions. Other researchers observed that marine triterpene glucosides such as frondoside A, cucumarioside A_2_-2, A_4_-2, and so on, had anticancer activity through different mechanisms, including increased expression of p21, p53, decreased expression of Bcl-2 and Mcl-1, increased expression of Bax, and inhibition of the noncovalent binding of topoisomerase 2α to DNA. Our group previously summarized their actions and their mechanisms [68]. Taken together, these suggest that marine triterpene glycosides may contribute to candidate anticancer agents. However, further studies on the relationship between the structure and the activity of these molecules are needed to improve the efficacy and safety of these compounds in treating leukemia patients.

### 4.3. Some Other Marine Inducers of Ceramide Accumulation

Several other marine natural products were found to be inducers of ceramide generation in tumor cells (Figure 2). Spisulosine (PharmaMar, ES-285), an anti-cancer agent isolated from the sea mollusk *Spisula polynema*, can cause tumor cell growth arrest or death. It was shown that spisulosine induces ceramide accumulation in prostate tumor cells [69]. This compound is under clinical testing [70]. The marine anhydrophytosphingosine, jaspine B, from the marine sponge *Jaspis* sp. inhibits the viability of murine B16 and human SK-Mel28 melanoma cells, increasing intracellular ceramide levels via the suppression of the activity of sphingomyelin synthase [71]. The marine lipopeptide somocystinamide A, from the cyanobacterium *Lyngbea majuscula*, a pluripotent inhibitor of angiogenesis and tumor cell proliferation, induces accumulation and aggregation of ceramide in treated cells, inhibiting leukemic Jurkat and CEM cells at as low IC_50_ as 3 and 14 nM [72].

## 5. Conclusions

Ceramide is known as a tumor suppressor lipid. It has been shown that some marine natural products and particularly stichoposides from sea cucumbers have antitumor activity through the generation of ceramide. STC, which generates ceramide by the activation of acid SMase and neutral SMase, results in inducing apoptosis of leukemia cell lines and inhibits the growth of leukemia xenografts. In contrast, STD induces apoptosis of leukemia cells and inhibits growth of leukemia xenografts through the activation of Fas/CerS6/p38 kinase. These findings suggest that the potency of stichoposides and molecular mechanisms underlying STC- and STD-induced apoptosis might be affected by a sugar attached to the aglycone of stichoposides. Thus, further understanding the structural characteristics regulating the biological activities of marine triterpene glycosides is essential when developing anticancer agents from natural marine products. It suggests the further search for new bioactive marine glycosides and other marine metabolites, which are promising anticancer agents and/or molecular instruments, regulating ceramide metabolism in tumor cells.
